# Cognitive Profiles of Developmental Dysgraphia

**DOI:** 10.3389/fpsyg.2018.02006

**Published:** 2018-11-22

**Authors:** Diana Döhla, Klaus Willmes, Stefan Heim

**Affiliations:** ^1^Department of Psychiatry, Psychotherapy and Psychosomatics, Medical Faculty, RWTH Aachen University, Aachen, Germany; ^2^Department of Neurology, Medical Faculty, RWTH Aachen University, Aachen, Germany; ^3^Institute of Neuroscience and Medicine (INM-1), Forschungszentrum Jülich, Jülich, Germany

**Keywords:** developmental dysgraphia, spelling, profiles, comorbidities, phonological processing, auditory processing, visual attention, visual magnocellular functions

## Abstract

Developmental dysgraphia is a disorder of writing/spelling skills, closely related to developmental dyslexia. For developmental dyslexia, profiles with a focus on phonological, attentional, visual or auditory deficits have recently been established. Unlike for developmental dyslexia, however, there are only few studies about dysgraphia, in particular about the variability of its causes. Research has demonstrated high similarity between developmental dyslexia and dysgraphia. Thus, the aim of the study was to investigate cognitive deficits as potential predictors of dysgraphia, analogously to those for dyslexia, in order to identify dysgraphia profiles, depending on the particular underlying disorder. Different tests were carried out with 3rd and 4th grade school children to assess their spelling abilities, tapping into phonological processing, auditory sound discrimination, visual attention and visual magnocellular functions as well as reading. A group of 45 children with developmental dysgraphia was compared to a control group. The results showed that besides phonological processing abilities, auditory skills and visual magnocellular functions affected spelling ability, too. Consequently, by means of a two-step cluster analysis, the group of dysgraphic children could be split into two distinct clusters, one with auditory deficits and the other with deficits in visual magnocellular functions. Visual attention was also related to spelling disabilities, but had no characteristic distinguishing effect for the two clusters. Together, these findings demonstrate that a more fine-grained diagnostic view on developmental dysgraphia, which takes the underlying cognitive profiles into account, might be advantageous for optimizing the outcome of individuum-centered intervention programs.

## Introduction

Developmental dysgraphia is a disorder characterized by difficulties in the acquisition of writing/spelling skills despite adequate schooling, visus and normal IQ. It is closely related to developmental dyslexia, a disorder of the acquisition of reading skills, which has been more in the focus of investigation for the past years. As defined by the American Psychiatric Association (2014) and the World Health Organisation [WHO] (2018) dyslexia and dysgraphia can co-exist as well as occur alone. The prevalence for reading and writing impairments is reported to be about 7–17% (Shaywitz and Shaywitz, 2005; Hawke et al., 2009).

There are several parallels between dyslexia and dysgraphia with respect to their underlying cognitive abilities and relevant cognitive skills (for a detailed review see Döhla and Heim, 2016), which shall be outlined here briefly. (1) There is evidence for a link between reading and spelling and *phonological processing* abilities. For instance, Snowling (2000) describes phonological awareness as the most known underlying deficit of developmental dyslexia. Phonological awareness as well as phonological working memory was reported to play an important role for dyslexia (Seigneuric and Ehrlich, 2005; Pennington et al., 2012) as well as for dysgraphia (Moll et al., 2009, 2012; Winkes, 2014; Capodieci et al., 2018 only for working memory). (2) The *automatization* of linguistic, motor and cognitive skills is supported by the cerebellum (Ito, 2008). Consequently, a dysfunctional cerebellum leads to problems with procedural learning resulting in a deficit in automatization that finally ends up in reading and writing deficits (dyslexia: e.g., Fawcett et al., 1996; Nicolson et al., 2001; Tiffin-Richards et al., 2001; dyslexia and dysgraphia: Nicolson and Fawcett, 2011; World Health Organisation [WHO], 2018). The relevance of automatization for reading and spelling, however, is not undisputed, since other studies failed to observe a deficit in a variety of automatization tasks for dyslexics (Heim et al., 2008 for children; Ramus et al., 2003 for adult). (3) There is ample evidence for the impact of *magnocellular functions* on reading, in particular *auditory processing skills* (Ramus et al., 2003; Steinbrink et al., 2014) and *visual processing* (Stein, 2001; Tholen et al., 2011). The connection of auditory processing and spelling has also been demonstrated (Schaadt et al., 2015). (4) Moreover, the relation between reading deficits and deficits in orienting spatial *attention* have been demonstrated, e.g., by Facoetti et al. (2003). Bosse et al. (2007) reported that both visual attention deficits as well as a phonological disorder can be associated with dyslexia, thus causing reading problems for different reasons. Banfi et al. (2017) investigated visuo-spatial cueing effects for children with isolated reading and spelling problems as well as a combined disorder. In contrast to children with an isolated reading or spelling disorder, children with a combined reading and spelling deficit showed a cueing deficit, which means, no significant difference in reaction time between valid and invalid cues. Dyslexic and dysgraphic children differed with respect to a position effect (Banfi et al., 2017). Whereas poor readers had a strong right-over-left advantage, poor writers had no position effect. Connecting visual and auditory information is crucial for learning to read and spell, e.g., with respect to grapheme–phoneme and, respectively, phoneme–grapheme correspondence. During speech perception, typically developing children profit from the bimodal presentation of stimuli: the combination of printed letters (visual stimulus) and speech sounds (auditory stimulus). Schaadt et al. (2018) revealed visual-auditory speech perception difficulties for children with spelling difficulties. Usually the combination of visual information and auditory processing supports information processing, but children with spelling deficits seem to fail in using this crossmodal integration (Schaadt et al., 2018). (5) Several studies revealed comorbidity between ADHD and spelling deficits (Adi-Japha et al., 2007; Capodieci et al., 2018). (6) Finally, the connection of SLD and later reading and writing performance has already been in the focus of investigation (dyslexia: Pennington and Bishop, 2009; dysgraphia: Puranik and Lonigan, 2012).

Because of the heterogeneity of diverse underlying deficits, a lot of research has been conducted to identify profiles of developmental dyslexia (e.g., Lachmann et al., 2005; Bosse et al., 2007; Reid et al., 2007). Heim et al. (2008) found three different dyslexic clusters: their Cluster A performed worse in phonological, visual and auditory tasks, Cluster B was characterized by a deficit only in phonological awareness and Cluster C scored worse only in visuospatial attention. Interestingly, automatization skills did not seem to have an influence on reading skills. Because of the known similarities of developmental dyslexia and developmental dysgraphia, it can be assumed that developmental dysgraphia might be characterized by meaningful profiles as well.

As indicated in Döhla and Heim (2016), the close relationship of dyslexia and dysgraphia as disorders on the one hand and the documented relationship between reading disability in dyslexia and deficits in the variables mentioned above (i.e., auditory processing, visual magnocellular functions and visual attention), on the other hand, show that the latter might also play a critical role for success or failure in acquiring spelling skills. The important influence of phonological processing on reading and spelling performance has already been established. But overall, there is much richer evidence for the field of dyslexia. Therefore the aim of the present study is to transfer existing knowledge about developmental dyslexia to dysgraphia with a focus on spelling abilities and consequently to investigate if differential profiles of developmental dysgraphia exist, and if so, whether these potential profiles also resemble those reported for dyslexia. Characterizing such profiles might help to specify prevention and therapy methods later on. For the sake of comparability to the previous study about cognitive profiles of dyslexia by Heim et al. (2008), the methodological approach of the study was kept as similar as possible: (1) The groups of dysgraphic and normal writers were compared against each other, with sample sizes in the present study which are comparable to those used by Heim et al. (2008) in order to identify three clusters. (2) To identify profiles, the group of dysgraphic children was clustered with respect to performance in tests of phonological processing, auditory sound discrimination, visual processing, and visual attention. In contrast to the previous study of Heim et al. (2008), scores for phonological working memory were entered into the study as a further variable, whereas automatization was excluded because it had not contributed to any of the dyslexia profiles (see also Ramus et al., 2003), and also in order not to exhaust children with too long testing sessions. The deficit profiles of these groups were then established by comparisons between the clusters obtained and between each of these clusters and the control group. (3) Finally, relationships between writing and reading were assessed. For the sake of simplicity, the comorbid abilities and skills are subsumed under the term “cognitive variables” in the remainder of this paper.

## Materials and Methods

### Participants

One hundred and thirty-two children and their parents agreed to participate. Out of these, those 98 children, who had a non-verbal IQ ≥ 70 and thus did not suffer from a general learning disorder according to the ICD-10; World Health Organisation [WHO] (2018), were included. Children with *T*-score < 37/percentile < 10 in a standardized German spelling assessment (see below) were assigned to the group of dysgraphic children. Children with a *T*-score ≥ 43 (i.e., percentile ≥ 25) were assigned to the group of normally spelling children. The *T*-scores of 37 and 43 were chosen because of non-perfect reliability of the test: The 90% confidence interval for the *T*-score 40 denoting the lower boundary of the normal value range is 40 ± 3.2. Out of the 132 children who volunteered, 25 did not fit the inclusion criteria. Moreover, data sets of nine children were not complete and thus could not be included in the analysis, yielding a total of 98 (65 boys and 33 girls) valid and complete data sets. Fifty-four children were in 3rd grade, 44 in 4th grade. An overview of the sample of participants is shown in Table 1. Twenty-one of the 45 dysgraphic children additionally had developmental dyslexia. We considered this issue by running separate analyses for the entire sample and only for the dysgraphic children with no reading difficulties, respectively (see below).

**Table 1:** Overview of the study participants.

	*n*	Age *M* (*SD*)	Non-verbal IQ *M* (*SD*)	Spelling (*T*-scores) *M* (*SD*)
Dysgraphics	45	9.9 (0.6)	91.6 (11.7)	31.1 (4.0)
Controls	53	9.9 (0.6)	108.3 (16.1)	51.7 (6.9)


The volunteers for this study had been recruited from six different German primary schools and special education schools from Cologne and Mönchengladbach, which agreed to take part, furthermore from one practice for speech therapy in Aachen, in the period between March 2014 and April 2015. Parents were provided detailed information about the content of the study according to the Declaration of Helsinki (World Medical Association, 2000). Written informed consent was obtained from all parents and children before participation. The study was approved by the local Ethics Committee of the Medical Faculty, RWTH Aachen University.

### Procedure

There were two test sessions. Spelling abilities, IQ and reading abilities were tested in a group setting on the 1st day. On the 2nd day, dysgraphic and non-dysgraphic children who fulfilled the inclusion criteria stated above were tested individually for their performance in phonological processing, auditory sound discrimination, visual magnocellular functions, and visual attention. The order of tests on the 2nd day was counterbalanced over participants to avoid order effects. All tests were administered in a quiet room in the schools or in the practice for speech therapy.

### Tests

The tests for spelling ability and IQ were administered to check inclusion criteria and are therefore described first. The other tests served as the dependent variables when investigating for clusters in the dysgraphic sample. An overview of the different tests and their settings is presented in Table 2.

**Table 2:** Overview of the different tests and their settings.

Domain	Test	Setting
Spelling	DRT-3/4	Group
Reading	KNUSPEL-L	Group
Non-verbal IQ	CFT-20-R Part 1	Group
Phonological awareness	BAKO 1-4 Subtest 4/6	Individual
Phonological working memory	Mottier	Individual
Auditory sound discrimination	H-LAD Subtest 1 a-c	Individual
Visual magnocellular functions	Moving star field	Individual – computerized
Visual attention	Posner paradigm	Individual – computerized


#### Spelling Ability

Spelling skills were tested with the German DRT-3 (Müller, 2003, for grade 3) or DRT-4^1^ (Grund et al., 2004, for grade 4). Sentences with a missing word were presented to the children and they were asked to write down the missing word, e.g., “Bert kauft das _______.” [“Buch”] (*“Bert buys the* _______.” *[“book”])*. This test provides *T*-scores for spelling accuracy.

#### Non-verbal Intelligence

Non-verbal intelligence was assessed with the CFT 20-R^2^ (Weiß, 2006). The test was administered in its short form (Part 1; with a reliability of 0.92) in order not to exhaust the children too much because of the long testing time (for test details see Heim et al., 2008). This test provides age-related IQ scores.

#### Reading Ability

Reading competence of participants was assessed with the KNUSPEL-L^3^ (Marx, 1998). Children had to perform four different tasks: Subtest 1, “Auditory comprehension” (German: “Hörverstehen”); subtest 2, “Recoding” (German: “Rekodieren”); subtest 3, “Decoding” (German: “Dekodieren”) and subtest 4, “Reading Comprehension” (German: “Leseverstehen”).^4^ Finally, the test provides two different norms, one for “precursor skills” which means basic skills which are considered necessary for learning to read (consisting of subtest 1–3) and one for reading performance (consisting of subtest 2–4), the latter entering the analysis. The test differentiates between monolingual and multicultural class norms (*T*-scores and percentile ranks) for grades 1 to 4, each in the middle or at the end of the school year. The tested classes hosted a variety of nationalities, so multicultural class norms were chosen.

#### Phonological Processing

##### Phonological awareness

The ability to work with the phonological structure of words like recognizing, segmenting, synthesizing and manipulating phonemes, syllables and onsets and rhymes was tested with the German test BAKO 1–4^5^ (Stock et al., 2003). Two subtests were chosen out of the set of seven subtests. Children had to do one receptive subtest, test 6: “Vowel length detection” (German: “Vokallängenbestimmung”), and one productive test, test 4: “Phoneme exchange” (German: “Phonemvertauschung”). In test 6: ”Vowel length,” participants had to identify one out of four acoustically presented pseudowords with a vowel length different from the other three pseudowords (e.g., “[mα:ϱ] – [Rα:s] – [dak] – [lα:t]”: [dak] is pronounced with a short vowel in contrast to the other three words). Test 4 “Phoneme exchange” requires children to change the first two phonemes of words and pseudowords which are presented auditorily, (e.g., /iftak/ → /fitak/). Separate norms for grades 1 to 4 were given for both tests. Because the scores of both tests were positively correlated in the previous study of Heim et al. (2008; *r* = 0.41; *p* < 0.001) and also in the present study (*r* = 0.306; *p* = 0.002), the average *T*-score was calculated for each child and used for further analyses as the measure for phonological awareness as in the Heim et al. (2008) analysis.

##### Phonological working memory

Phonological working memory was tested with the “Mottier-Test” (Mottier, 1951). Children were asked to repeat 30 sequences of meaningless syllables (e.g., “lu-ri” or “bi-ga-do-na-fe-ra”). The new standardization by Wild and Fleck (2013) for children aged between 5 and 17 years is valid for both mono- and bilingual children and thus their *T*-scores constituted the basis for the analysis.

#### Auditory processing

The values of subtest 1 from the H-LAD^6^ (Brunner et al., 2005) were included in the analysis. Children had to determine whether pairs of real words or syllables were equal or different (e.g., [k℧s] – [g℧s], [bα:] – [bα:], [kεm∂n] – [kεn∂n]). *T*-scores and percentile ranks for grades 1–4 are provided. For the present analysis, the *T*-scores for 3rd and 4th graders were used.

#### Visual Magnocellular Functions

In the computerized paradigm “Star field” (Wilms et al., 2005) children saw a moving random dot pattern and had to click the left mouse button as quickly as possible when its motion changed. The dot pattern was changing its motion (expanding, static and contracting) after a varying time interval of 1–3 s (for details see Wilms et al., 2005). Motion direction as well as time intervals were pseudo-randomized. The average reaction time was used for subsequent analysis.

#### Visual Attention

In the Posner Paradigm (Posner, 1980; Vossel et al., 2006) the participants had to click the left or right mouse button as quickly as possible, to indicate on which side of the screen a target stimulus is shown, to measure the participant’s reaction time. In advance of each trial, in the middle of the screen a neutral, a valid or invalid cue or no cue appears. The neutral cue indicates that a target stimulus will appear and thereby prepares the participant that a response is to be expected soon. Other than the neutral cue, valid and invalid cues point to a particular side. The valid cue points to the side where the target stimulus will appear and therefore is helpful and informative in order to be able to push the button faster. The invalid cue points to the opposite side of the subsequent target stimulus. It is therefore misleading and the participant has to shift the focus of attention back to the correct target-side before pushing the button. Two values for aspects of visual attention, the alertness effect and the CVE, were included in the analysis. *Alertness* is the general readiness of the brain to respond to an expected stimulus (Wiegand et al., 2017), calculated as the reaction time difference between average reaction time of no cue trials versus neutral trials which contain a cue alerting the subject to an upcoming stimulus, but without directional information where that stimulus is going to appear on the screen. The CVE is computed as the reaction time difference between invalidly cued and validly cued trials. This difference indicates how quickly attention can be shifted from one location toward a new location. Smaller CVE values indicate quicker and more effective reorienting of attention.

All computerized tests were programmed and administered with Presentation^®^ (version 0.7, Neurobehavioral Systems, Albany, CA, United States) run on an Acer Travelmate 5744 laptop under Windows 7.

### Data Analysis

Only participants with complete data sets were included in the data analysis (*n* = 98) using SPSS 22 for Mac IOS (SPSS Inc., Chicago, IL, United States). To ensure comparability, analysis in the present study was very similar to the analysis of the previous study about cognitive profiles of dyslexia (Heim et al., 2008). In that study, first a general comparison of the two groups (dyslexic children vs. normally spelling children) was conducted. A partitioning cluster analysis for the group of impaired children was done next and followed by discriminant analyses. In the present study, a similar procedure was chosen, as explained in the following paragraphs.

#### Discriminant Analysis Part 1

In a first step, all children were compared with a linear discriminant analysis to find out which of the six variables considered (phonological awareness, phonological working memory, auditory sound discrimination, magnocellular function and visual attention: CVE and alertness) allow for the best separation of the whole dysgraphic group and the group of normally spelling children. We chose the discriminant analyses instead of a series of separate two-sample *t*-tests because the former, rather than the latter, consider potential covariation of the dependent variables in the analysis.

#### Two-Step Cluster Analysis

Next, a two-step cluster analysis was conducted to identify the optimum number of profiles in the dysgraphic sample. As previously done by Heim et al. (2008), the analysis was run with the following specifications: maximum number of clusters: 15, distance estimation: log-likelihood, clustering criterion: Akaike’s information criterion, outlier treatment: no noise-handling, initial distance change threshold: 0, depth levels: a maximum of three. All variables were standardized during the clustering procedure.

#### Discriminant Analyses Parts 2 to 4

A series of linear discriminant analyses followed. The clusters were compared directly with each other and also with the control group. For all discriminant analyses the following settings were selected (in line with the procedure used by Heim et al., 2008): the dependent variables were entered step-wise, inclusion criterion: *p* ≤ 0.05, exclusion criterion: *p* ≥ 0.10. Priors were set equal. Wilks’ lambda was calculated for each step. For the assignment of children to a particular group the leaving-one-out method was used to prevent biased (under-) estimates of misclassification rates.

#### Additional Analysis: The Relationship of Reading and Spelling Skills

Next, several *chi*-square analyses were conducted in order to test for distributional differences of reading impairment among the dysgraphic participants as well as for sex and grade differences across the clusters and among dysgraphics and normally spelling children. For age, a *t*-test for independent samples was carried out in order to assess mean age differences between dysgraphics and normally spelling children.

Furthermore, a series of *t*-tests for independent samples was run in order to compare the reading competence of the dysgraphic children in both clusters with each other and with that of the normally spelling children. In addition the effect sizes were calculated with Cohen’s *d* (Lenhard and Lenhard, 2016) for the average reading competence of Clusters 1 and 2 in comparison to normally spelling children.

Finally, the observed cluster solution from the two-step cluster analysis, which included data from all dysgraphic children (with and without reading deficits) was revalidated including only those dysgraphic children with no diagnosed reading deficits. To this end, the same parameter settings were used for a pre-defined 2-cluster solution. The coincidence in assignment of children with pure dysgraphia to the original clusters and the newly established clusters in that second analysis is reported in a 2 × 2 contingency table.

## Results

### Cognitive Variables: Group Differences of Dysgraphic vs. Normally Spelling Children

The first discriminant analysis was employed to compare the whole group of dysgraphic children with the normally spelling children with respect to six dependent variables using a stepwise forward selection approach to find the best discriminating variables. The two groups differed significantly in phonological processing: in phonological working memory (Wilks’ λ = 0.57; *p* < 0.001) as well as in phonological awareness (Wilks’ λ = 0.66; *p* < 0.001). Thirty-eight of the 45 dysgraphic children (84.4%) and 41 of the 53 normally spelling children (77.4%) were correctly assigned to their spelling skill groups on the basis of the set of selected cognitive variables using the leaving-one-out method, resulting in a total positive classification rate of 80.6% on the basis of the two variables for phonological processing. Table 3 gives an overview, presenting means and standard deviations for *T*-Scores and raw scores of the participants’ performance in the cognitive variables.

**Table 3:** Means (M) and standard deviations (SD) of the two Clusters and Controls for the cognitive variables.

	Cluster 1 (*n* = 17)		Cluster 2 (*n* = 28)		Cluster 1 + 2 (*n* = 45)		Controls (*n* = 53)	
	*M*	*SD*	*M*	*SD*	*M*	*SD*	*M*	*SD*
**Spelling (DRT 3/4)**								
*T*-Score	31.70	4.72	30.71	3.58	31.08	4.02	51.68	6.88
Raw score grade 3	37.14	4.35	39.82	2.87	38.78	15.10	20.11	8.34
Raw score grade 4	9	6.56	13.17	1.47	11.78	4.06	32.29	5.37
**Phonological awareness (BAKO)**								
*T*-Score	40.82	5.17	39.96	3.20	40.29	4.02	49.71	8.13
Raw score	3.71	1.87	3.53	1.28	3.6	1.51	7.08	2.47
**Phonological working memory (Mottier)**								
*T*-Score	42.32	11.02	40.03	9.75	40.89	10.19	54.52	10.51
Raw score	15.34	4.42	14.43	4.20	14.73	4.26	20.47	4.49
**Auditory sound discrimination (H-LAD)**								
*T*-Score	56.35	5.34	43.86	11.82	48.58	11.56	56.47	8.29
Raw score	23.18	1.34	18.57	5.87	20.31	5.19	23.30	2.22
**Visual magnocellular function (Starfield)**								
Reaction time	1111.91	160.55	824.57	166.35	933.12	214.95	801.30	182.61
**Visual attention (Posner paradigm)**								
CVE: reaction time	100.39	152.28	61.33	67.39	76.08	107.64	73.04	58.71
Alerting: reaction time	86.39	155.64	42.97	62.56	59.38	108.0	40.38	65.70
**Reading competence (KNUSPEL-L)**								
*T*-Score	35.18	9.59	38.57	8.48	37.29	8.96	51.38	8.90
Raw score	113.47	24.40	121.5	20.60	118.47	22.19	153.62	22.04


### Cognitive Clusters of Dysgraphia

**FIGURE 1:**
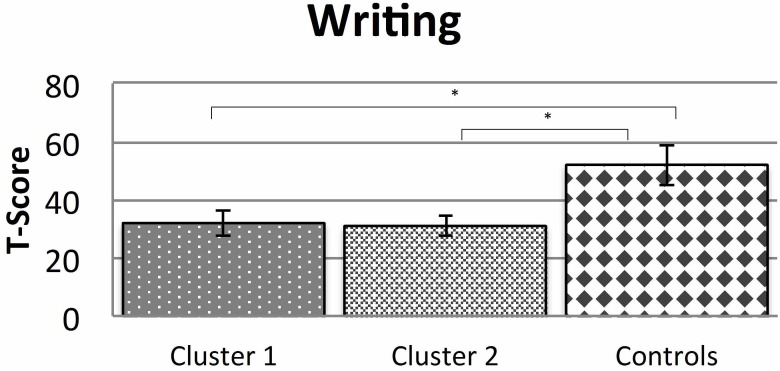
Spelling skills (DRT 3/4) in the two clusters of dysgraphic children and the normally spelling children (mean and SD; ^∗^*p* < 0.05).

The cluster analysis of the dysgraphic group based on the set of six cognitive variables (phonological awareness, phonological working memory, auditory sound discrimination, magnocellular function and visual attention: CVE and alertness) yielded two clusters (Cluster 1: *n* = 17; Cluster 2: *n* = 28). The average *T*-Scores for spelling competence of the clusters and of the normally spelling group are displayed in Figure 1.

In the subsequent discriminant analyses, the comparison of Clusters 1 and 2 revealed that auditory sound discrimination (Wilks’ λ = 0.33; *p* < 0.001) and visual magnocellular functions (Wilks’ λ = 0.57; *p* < 0.001) out of the profile of six variables contributed significantly to discrimination among both clusters. Cluster 1 could be identified as being significantly worse in visual magnocellular functions, Cluster 2 scored significantly worse in auditory sound discrimination – separated clusters with 16/17 (94.1%) correctly identified dysgraphic children in Cluster 1 and 27/28 (96.4%) in Cluster 2 with an overall correct assignment of 95.6%.

In a next step, Cluster 1 was compared to the normally spelling children. Cluster 1 differed from the normally spelling children in phonological working memory (Wilks’ λ = 0.55; *p* < 0.001), and visual magnocellular functions (Wilks’ λ = 0.63; *p* < 0.001). The overall rate of correct classifications was 91.4%, with 50/53 (94.3%) of the normally spelling children and 14/17 (82.4%) of the dysgraphic children in Cluster 1 correctly assigned.

Children in Cluster 2 and control children differed in phonological working memory (Wilks’ λ = 0.51; *p* < 0.001), auditory sound discrimination (Wilks’ λ = 0.58; *p* < 0.001) and phonological awareness (Wilks’ λ = 0.68; *p* < 0.001) with an overall classification rate of 87.7% (48/53 = 90.6% for the normally spelling children and 23/28 = 82.1% of the dysgraphic children of Cluster 2 correctly assigned to their groups).

**FIGURE 2:**
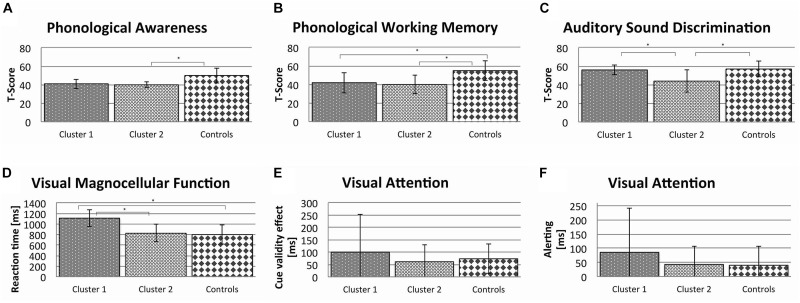
Comparison of the dysgraphic clusters and the group of normally spelling children with respect to the different cognitive variables displayed with *T*-Scores for **(A–C)** and reaction time in ms for **(D–F)**, thus higher values in the visual tests indicate longer reaction times (linear discriminant analysis with mean and SD; ^∗^*p* < 0.05).

Figure 2 shows the average *T*-scores for phonological awareness, phonological working memory, auditory sound discrimination and the average reaction time (higher reaction times indicating worse performance) for visual magnocellular functions and visual attention separately for each cluster and the control group.

The two variables for visual attention revealed no significant differences between the clusters (Alerting: Wilks’ λ = 0.31; *p* = 0.114; CVE: Wilks’ λ = 0.32; *p* = 0.341) and the normally spelling children (Cluster 1 vs. normally spelling children: Alerting: Wilks’ λ = 0.52; *p* = 0.517; CVE: Wilks’ λ = 0.54; *p* = 0.551 and Cluster 2 vs. normally spelling children: Alerting: Wilks’ λ = 0.51; *p* = 0.602; CVE: Wilks’ λ = 0.51; *p* = 0.492).

**FIGURE 3:**
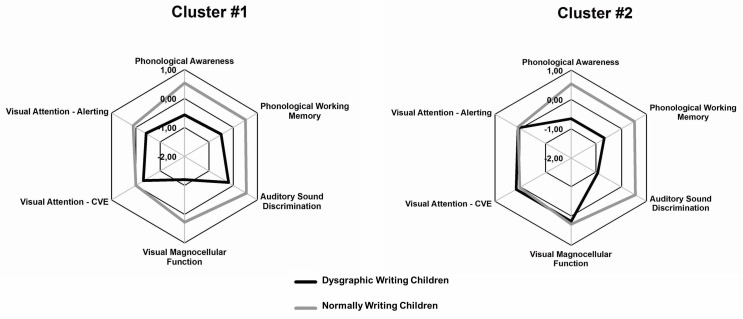
Fingerprints of the two dysgraphic clusters in comparison to normally spelling children, a z-transformation was conducted and a reversal of signs (positive/negative) was done if necessary so that positive *z* scores consistently represent good performance.

Figure 3 shows the differing average profiles of the dysgraphic clusters, displayed as fingerprint plots for the six chosen cognitive variables.

Since the discriminant analysis revealed significant differences from controls in phonological awareness only for Cluster 2 but not for Cluster 1, an additional *t*-test was conducted to validate the results also reporting Cohen’s *d* and the statistical power estimate. Cluster 1 (*t*_43.09_ = 5.29, *p* < 0.001 ^7^, *d* = -1.31, *power estimate* = 0.996) and Cluster 2 (*t*_74.63_ = 7.68, *p* < 0.001, *d* = -1.58, *power estimate* = 0.999999) showed significant differences in comparison to normally spelling children after Bonferroni-correction. The comparison of Clusters 1 and 2 revealed no mean difference (*t*_23.56_ = 0.62, *p* = 0.543, *d* = -0.2 *power estimate* = 0.1) and thus confirmed and extended the previous results: phonological awareness deficits are a common factor for both dysgraphia clusters (as expressed in the significant *t*-tests) but explain independent variance to a different degree (as expressed in the only partly significant solutions of the discriminant analyses).

The chi-square analyses revealed no sex and grade differences across the clusters (sex: Pearson’s *chi*_1_^2^ = 0.37, *p* = 0.546, grade: Pearson’s chi_1_^2^ = 0.95, *p* = 0.758). The comparison of the whole group of dysgraphic vs. normally spelling children also revealed no sex differences (Pearson’s *chi*_1_^2^ = 3.48, *p* = 0.18) and no age differences (*t*_96_ = -0.433, *p* = 0.666).

### The Relationship of Reading Ability and Dysgraphia

**FIGURE 4:**
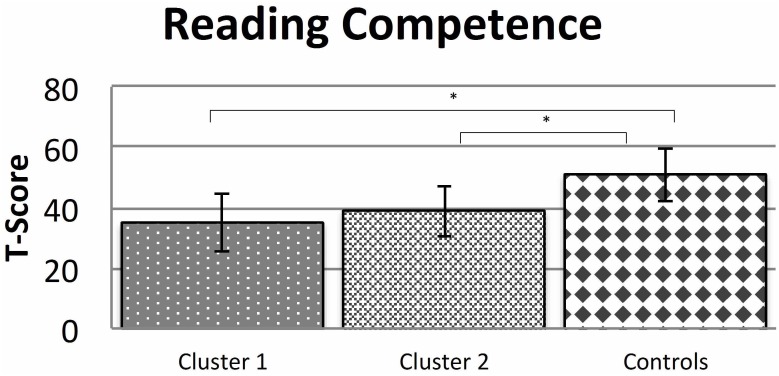
Reading skills (Knuspel-L; mean and SD; ^∗^*p* < 0.05) of the children in the dysgraphic clusters and the normally spelling children.

In an additional analysis, we tested whether the actual degree of reading competence differed across the clusters and the normally spelling children. Mean and standard deviation of the reading skills of the two dysgraphic clusters and the normally writing children are displayed in Figure 4. For this purpose we ran a series of *t*-tests for independent samples, comparing pairwise the reading scores of the children in the two clusters and normally spelling children. The results of these *t*-tests revealed that the co-occurrence of developmental dyslexia with dysgraphia does not seem to have a substantial effect on the formation of the dysgraphia clusters in the present sample. The comparison of Cluster 1 vs. Cluster 2 provided no significant differences (*t*_43_ = -1.24, *p* = 0.222). In contrast, however, comparison of each cluster against normally spelling children revealed significant mean differences also after Bonferroni-correction: Normally spelling children vs. Cluster 1 (*t*_68_ = 6.41, *p* < 0.001) and normally spelling children vs. Cluster 2 (*t*_79_ = 6.26, *p* < 0.001).

In a next step, the average scores for the variable reading competence of the two clusters versus normally spelling children were compared and revealed very high effect sizes (Field, 2014): Cluster 1 vs. normally spelling children (Cohen’s *d* = -1.75) and Cluster 2 vs. normally spelling children (Cohen’s *d* = -1.47).

Finally, re-running the two-step cluster analysis to study the assignment of the purely dysgraphic children with no reading difficulties (*n* = 27 instead of *n* = 45) to the two clusters revealed that most children were assigned to the same clusters (95% for cluster 2 and 71.4% for cluster 1; cf. Table 4). Together, these results further corroborate the analyses above, indicating that reading performance had no substantial effect on the cluster structure in the present sample.

**Table 4:** Assignment of purely dysgraphic children (*n* = 27) to the two clusters in the original two-step cluster analysis (*n* = 45) and the 2-cluster replication (*n* = 27).

	New analysis (*n* = 27)				
		Cluster 1	Cluster 2	Total	% overlap
**Original analysis (with *n* = 45)**	**Cluster 1**	5	2	7	71.4
	
	**Cluster 2**	1	19	20	95.0

## Discussion

The aim of this study was to identify cognitive deficit profiles of developmental dysgraphia depending on the underlying disorders. In a further step, the new evidence about diverse patterns of impairment in developmental dysgraphia was compared to the existing knowledge about developmental dyslexia to point out communalities and differences.

The present study provided evidence that there are three important cognitive abilities that may differentially characterize dysgraphia: phonological, auditory and visual magnocellular processing. Based on assessment procedures for these types of processing abilities, two distinct clusters of children with different cognitive profiles could be identified. Whereas phonological awareness and phonological working memory are general characteristics for developmental dysgraphia, distinguishing the children from normal writers, auditory processing and visual magnocellular functions were identified as differentiating variables, distinguishing dysgraphic children from normally spelling children as well as the two dysgraphic clusters from each other, rather unrelated to their skills in reading performance. These results will now be discussed in detail.

### Underlying Cognitive Skills of Developmental Dysgraphia

In a first step, we examined for which cognitive variables the whole group of dysgraphic children differed from normally spelling children. Phonological processing skills, i.e., phonological awareness and phonological working memory emerged as significantly differentiating variables distinguishing dysgraphic from normally spelling children in 80.6%. This confirms the earlier investigation that performance in phonological processing is an important variable for dysgraphia (phonological awareness: e.g., Moll et al., 2009; phonological working memory: e.g., Steinbrink and Klatte, 2008; Steinbrink et al., 2008; Winkes, 2014 with only an indirect influence of phonological working memory on phonological awareness, which consequently influences spelling competence). The other cognitive variables (auditory processing, visual magnocellular function and visual attention) did not distinguish the whole group of dysgraphic children from the normally spelling children.

### Profiles of Developmental Dysgraphia

However, the two-step cluster analysis went beyond this initial finding. It revealed structure within the group of dysgraphic children, separating them into two clusters. Two clearly distinguishable profiles of dysgraphic children appeared based on visual-magnocellular vs. auditory processing abilities. In comparison to normally spelling children, besides the already documented dysfunction in phonological working memory, Cluster 1 was characterized by deficits in visual magnocellular function, alongside a numerical but non-significant reduction also in visual attentional processing. In contrast, Cluster 2 was characterized by significantly worse auditory performance in comparison to normally spelling children, with significant deficits in both variables representing phonological processing abilities (phonological awareness and phonological working memory). The direct comparison of the two clusters revealed the differential impairment pattern in these profiles, with Cluster 1 demonstrating a visual impairment and Cluster 2 an auditory impairment.

Even after excluding dyslexic children, the two clusters remained similar although the Cluster 1 group was reduced more extensively with 7 dysgraphic children left in contrast to the Cluster 2 group with 20 dysgraphic children remaining. After excluding dyslexic children 6 children fall in Cluster 1 and show a visual magnocellular deficit and 21 in cluster 2 with an auditory deficit. This leads to the conclusion that even if visual magnocellular functions play a role for spelling, auditory functions seem to influence dysgraphic children more often. The exact role auditory and visual functions play throughout the course of literacy development needs to be addressed further in longitudinal studies.

In conclusion, the present study revealed new evidence that children with developmental dysgraphia are not homogeneous and that diverse cognitive variables, i.e., phonological awareness, phonological working memory, auditory processing and visual magnocellular function (with some visual attention problems) are important for developmental dysgraphia. These findings show that spelling is a complex process, influenced by diverse cognitive variables, which should be taken into account for diagnosis and remediation. Usually only whole word and letter-by-letter reading/spelling are in the focus during the process of diagnosing dyslexics/dysgraphics. On the basis of the present data, inclusion of additional variables equivalent to those chosen for the study could be included in prediction and prevention programs. The question about the connection of developmental dysgraphia and dyslexia as well as their communalities and differences remains still unanswered and will be discussed below.

### Cognitive Profiles of Dysgraphia and Their Relationship to Dyslexia

The distributions of dysgraphic participants with vs. without accompanying reading difficulties across the clusters did not differ significantly in the present sample. These data can be taken to reflect either that the type of cognitive profile of a dysgraphic child is not influenced by his/her reading ability – or that the observed cognitive profiles have rather comparable impact on reading ability, at least in the sample studied here.

In order to reflect the communalities of developmental dysgraphia and dyslexia, the new evidence about dysgraphic children will now be compared to the existing knowledge about dyslexia. In a first step, the underlying skills of the spelling and reading deficit are compared and in a second step, the clusters obtained in the present study are compared to those previously found by Heim et al. (2008).

The present study corroborated the assumption that dyslexia and dysgraphia share several commonly underlying disorders (Döhla and Heim, 2016): Problems in phonological, auditory, and visual processing. Impaired phonological processing, already known to be closely related to dyslexia (phonological awareness deficit: e.g., Snowling, 2000; Steinbrink et al., 2008; Pennington et al., 2012 and phonological working memory deficit: e.g., Seigneuric and Ehrlich, 2005; Steinbrink et al., 2008), has also been investigated as an important characteristic for dysgraphia (phonological awareness deficit: e.g., Moll et al., 2009; phonological working memory deficit: e.g., Steinbrink and Klatte, 2008; Steinbrink et al., 2008; Winkes, 2014) and was also found by us. Also, there is much evidence that dyslexics can have deficits in auditory processing (Ramus, 2003; Steinbrink et al., 2014 for children and Ramus et al., 2003; Christmann et al., 2015 for adults). The present study has shown similar results for dysgraphic children: The clusters found reflect that auditory processing is impaired in some dysgraphic children but not in others. Besides their phonological processing deficits, children in Cluster 2 also yielded worse auditory performance in comparison to normally spelling children. With respect to the literature there are two scenarios: On the one hand, deficits in auditory processing and phonological awareness can appear independently, on the other hand severe auditory deficits can cause deficits in phonological skills (i.e., phonological awareness, phonological working memory, or rapid naming) and therefore affect reading as well (Ramus et al., 2003). In the present study, auditory processing deficits usually seem to appear together with deficits in phonological processing (here: phonological awareness and phonological working memory). Children in Cluster 2 scored worse on average than normally spelling children in auditory processing as well as phonological awareness and phonological working memory; conversely, however, deficits in phonological awareness did not necessarily occur together with deficits in auditory processing. The whole group of dysgraphics scored significantly worse than normally spelling children in phonological awareness and phonological working memory, but not all of them also had worse results in auditory processing. Dividing the whole group of dysgraphic children into clusters, the children in Cluster 1 were, besides visual deficits, characterized by impaired phonological working memory, but on average did not show worse results in auditory processing. Consequently, the data indicate that phonological processing deficits may not always be a result of auditory deficits.

Apart from phonological and auditory processing deficits, visual deficits have also been discussed as relevant for reading and dyslexia. Stein (2001), Heim et al. (2008) and Tholen et al. (2011) showed a connection of visual magnocellular processing and reading competence. The results of the present study revealed that visual magnocellular functions can be impaired in poor writers as well. Cluster 1 was characterized by deficits in visual magnocellular function. The neurobiological model (Ramus, 2004) describes a phonological deficit as the main cause for dyslexia, sometimes co-occurring with deficits in magnocellular functions. Stein (2001) in his general magnocellular theory argues for the reverse relationship: he sees the reason for dyslexia in a general magnocellular deficit that causes diverse cognitive deficits. Findings of Heim et al. (2008, 2010) supported the theory of Ramus (2004) in that phonological awareness is not depending on magnocellular processing skills. For dysgraphia, a visual magnocellular dysfunction may lead to a possible deficit of dysgraphic children, which occurs in some cases but not in others, just like deficits in auditory processing.

Finally, visual attention requires additional consideration. In contrast to dyslexics (e.g., Facoetti et al., 2003; Bosse et al., 2007), significantly worse performance in visual attention (CVE and alertness) has not been shown for dysgraphic children. But even if the numerical trend did not reach significance, the variable visual attention revealed interesting results nevertheless, because Cluster 1, the cluster with deficits in visual magnocellular function, also had numerically worse results in visual attention than Cluster 2 and the normally spelling children. With respect to visual attention skills, similarities to results by Banfi et al. (2017) can be pointed out, who investigated visuo-spatial attention skills in dyslexic and dysgraphic children. In their study, a difference between poor readers and writers was also detected with respect to a right-over-left advantage (position effect) for dyslexics and no position effect for dysgraphic children.

In summary, the present study revealed communalities with respect to deficits in underlying cognitive abilities of developmental dyslexia and dysgraphia, i.e., in phonological, auditory and visual magnocellular processing. The whole group of dysgraphic children in our study and dyslexics (in the study of Heim et al., 2008) alike differ from normally spelling/reading children with respect to worse performance in phonological processing (i.e., phonological awareness and phonological working memory), dyslexics additionally differ in worse performance in visual attention tasks (Heim et al., 2008).

Even if there are different profiles of dyslexic and dysgraphic children, developmental dyslexia and dysgraphia show similarities, although the variables were not completely identical in the study by Heim et al. (2008) and in the present study. Generally speaking, comparing profiles makes sense for both disorders and helps to characterize the disorders in a more fine-grained way. Examined in more detail, Heim et al. (2008) reported three distinct profiles of developmental dyslexia: Cluster A showed worse phonological, auditory and magnocellular skills, Cluster B only scored worse in phonological awareness tasks, Cluster C had impaired visual attention skills. The cluster analyses for dysgraphic children revealed Cluster 1 with deficits in visual magnocellular functions and Cluster 2 with deficits in auditory sound discrimination, both sharing deficits in phonological processing. Although partially different variables led to differentiating the children into different profile groups, visual and auditory skills contribute to the characterization of the clusters found for both disorders. In contrast to the dyslexic clusters in Heim et al. (2008), children with developmental dysgraphia could be distinguished as either showing auditory or visual disorders. Moreover, impaired phonological processing was generally characteristic both for dysgraphia and dyslexia in contrast to normally spelling/reading children, but was furthermore identified as a differentiating variable between clusters only for dyslexia.

The findings of the present study suggest that developmental dyslexia and dysgraphia have a common basis: they (1) share the fact that they have diverse underlying deficits and they (2) also share almost all of those deficits; they (3) moreover share the fact that impaired children can be subdivided into profiles and they (4) furthermore have in common that either impaired visual magnocellular functions or impaired auditory processing differentiate between dyslexic and dysgraphic children. With a closer look they also show differences in (5) the combination of those underlying deficits and finally (6) might differ with respect to possible profile groups. Thus, developmental dysgraphia and developmental dyslexia might be regarded as similar but not homologous with respect to their underlying cognitive profiles.

Future research should include a broader diagnostic assessment approach, comprising a large pool of different functions assessed to investigate possible further dysgraphic profiles. Furthermore it would be inspiring to substantiate the new evidence with imaging techniques and thus get more precise information especially of the magnocellular system and the cerebellum with respect to visual and auditory processing. A more fine-grained analysis by contrasting exclusively dyslexic and exclusively dysgraphic children in comparison to children with a combined disorder of reading and spelling skills may reveal interesting information with respect to underlying additional deficits and eventually question the actually supposed communalities of the two disorders.

## Conclusion

The present study revealed new insights about underlying deficits and possible performance profiles of developmental dysgraphia and communalities of them with developmental dyslexia. In comparison to normally spelling children, dysgraphic children score worse in phonological processing skills, i.e., phonological awareness and phonological working memory. Based on six variables, measuring cognitive abilities and acquired skills, dysgraphic children could be subdivided into two profiles, one with an auditory and phonological processing (phonological awareness and phonological working memory) deficit and another with an impairment in visual magnocellular functions and phonological working memory. In summary, the present study revealed evidence for underlying deficits of developmental dysgraphia, i.e., phonological and auditory processing impairments as well as deficits in visual magnocellular functions. Finally, a comparison revealed that developmental dysgraphia and dyslexia are similar but not homologous. They share a common basis with different individual characteristics. As a consequence, it is reasonable to transfer this new evidence about impaired underlying functions to therapeutic everyday practice and conduct a more fine-graded diagnosis of dysgraphic children and consequently tailor remediation on the basis of the patient’s individual resources or barriers.

## Author Contributions

DD: data acquisition, first manuscript draft, data analysis. DD, SH, and KW: concept, data analysis strategy, interpretation of results, revision of the manuscript drafts, and visualization of results.

## Conflict of Interest Statement

The authors declare that the research was conducted in the absence of any commercial or financial relationships that could be construed as a potential conflict of interest.

## Footnotes

1 DRT: Engl.: Diagnostic spelling test for 3rd/4th grade, German: Diagnostischer Rechtschreibtest für 3./4. Klassen.

2 CFT 20-R: Engl.: Cattell Culture Fair Test 20 – Revision, German: Grundintelligenztest Skala 2 - Revision.

3 KNUSPEL-L: Engl.: Knuspel’s reading exercises, German: Knuspels Leseaufgaben.

4 All tasks were about the little, fictive “Knuspel” creatures that lead children through the test. In the first subtest, children had to listen to questions and give answers in written form, e.g., “How many bad-tempered Knuspels and Knuspels in a good mood do you see on the previous page? Put a cross in the first box for each Knuspel.” In subtest 2, children had to recognize homophones (e.g., German “mehr” [meϱ] and “Meer” [meϱ]) or non-homophones (e.g., “Stahl” [∫ tα:l] and “Stall” [∫ tal]). Subtest 3 required the children to identify pseudo-homophones, i.e., indicate if words written incorrectly would sound like a real word if they were read aloud (e.g., “SANDT” [zant] sounds like “Sand” [zant] (*sand*) or “ROTT” [R⊃t] which only reminds of the German word “rot” [Ro:t] (*red*) but is not pronounced equally. Subtest 4 is similar to subtest 1 but this time, in order to test children’s reading comprehension, they had to read and answer the instruction by themselves.

5 BAKO 1–4: Engl.: Basic competences for reading and spelling skills, German: Basiskompetenzen für Lese-Rechtschreibleistungen.

6 H-LAD: Engl.: Heidelberger test for auditory sound discrimination, German: Heidelberger Lautdifferenzierungstest.

7 *p*-values < 0.05 are reported already taking into account Bonferroni-correction, i.e., the alpha level to be compared with the *p*-value is already divided by the number of comparisons.

## Abbreviations

BAKO 1–4, Engl.: Basic competences for reading and spelling skills
German: Basiskompetenzen für Lese-Rechtschreibleistungen
CFT 20-R Engl.: Cattell Culture Fair Test 20 – Revision
German: Grundintelligenztest Skala 2 - Revision
CVE: cue validity effect
DRT, Engl.: Diagnostic spelling test for 3rd/4th grade
German: Diagnostischer Rechtschreibtest für 3./4. Klassen
DSM-V: diagnostic and statistical manual of mental disorders, the fifth edition
H-LAD, Engl.: Heidelberger test for auditory sound discrimination
German: Heidelberger Lautdifferenzierungstest
ICD-10: International Statistical classification of diseases and related health problems-tenth revision
KNUSPEL-L, Engl.: Knuspel’s reading exercises
German: Knuspels Leseaufgaben
WHO: World Health Organization
